# Peritoneal Sarcomatosis Secondary to Conventional Chondrosarcoma: A Case Report

**DOI:** 10.7759/cureus.103682

**Published:** 2026-02-15

**Authors:** Dorian Y Garcia-Ortega, Miguel A Clara-Altamirano, Claudia H Caro Sanchez, Ana P Melendez-Fernandez, Gabriela C Alamilla-Garcia, Andrea Góngora Nava, Cherilynn Martínez Valdovinos, Hugo R Domínguez-Malagón, Kuauhyama Luna-Ortiz

**Affiliations:** 1 Skin and Soft Tissue Sarcoma and Bone Tumors, National Cancer Institute, Mexico City, MEX; 2 Orthopaedic Oncology, National Cancer Institute, Mexico City, MEX; 3 Pathology, National Cancer Institute, Mexico City, MEX; 4 Surgical Oncology, National Cancer Institute, Mexico City, MEX; 5 Oncology, National Cancer Institute, Mexico City, MEX; 6 General Practice, National Cancer Institute, Mexico City, MEX; 7 Anatomic Pathology, National Cancer Institute, Mexico City, MEX; 8 Head and Neck, National Cancer Institute, Mexico City, MEX

**Keywords:** conventional chondrosarcoma, cytoreductive surgery, internal hemipelvectomy, peritoneal sarcomatosis, spontaneous tumor rupture

## Abstract

Chondrosarcoma is one of the most common primary malignant bone tumors and typically metastasizes hematogenously, most frequently to the lungs. Peritoneal dissemination is an exceptional presentation, usually associated with dedifferentiated subtypes or tumor rupture, and lacks standardized management strategies. We report the case of a 27-year-old male who presented with a large pelvic-abdominal mass and severe left lower-limb edema. Imaging revealed a large calcified tumor arising from pelvic zone III. Biopsy suggested a grade 1 conventional chondrosarcoma. The patient underwent an intraperitoneal surgical approach, during which spontaneous tumor rupture, hemoperitoneum, and peritoneal implants were identified. Complete macroscopic cytoreduction (CC-0) was achieved through type III internal hemipelvectomy, omentectomy, and resection of peritoneal implants. Final pathology confirmed grade 2 conventional chondrosarcoma with peritoneal involvement. The postoperative course was uneventful. The patient is currently receiving individualized adjuvant systemic therapy and shows no evidence of disease at early follow-up. Peritoneal sarcomatosis secondary to conventional chondrosarcoma is exceedingly rare. This case highlights that aggressive surgical management may be feasible in carefully selected patients and underscores the importance of multidisciplinary care in specialized sarcoma centers.

## Introduction

Chondrosarcoma is a malignant neoplasm of cartilaginous origin and is the second most common primary malignant bone tumor, accounting for approximately 20-30% of malignant bone tumors [1]. Its biological behavior is heterogeneous, primarily associated with histological grade. More than 85-90% are conventional low- or intermediate-grade chondrosarcomas, characterized by slow growth and low metastatic potential [2]. Only 5-10% of conventional chondrosarcomas are grade 3, which carry a significantly higher risk of metastasis and a worse prognosis [3].

Peritoneal sarcomatosis is defined as the multifocal spread of tumor implants on the peritoneal surface and is classically observed in sarcomas of intra-abdominal origin, such as leiomyosarcomas, liposarcomas, and gastrointestinal stromal tumors [4]. In contrast, peritoneal involvement in chondrosarcoma is exceptional, as this tumor metastasizes predominantly hematogenously, with a marked predilection for the lungs [2,5].

The available literature on peritoneal dissemination in chondrosarcoma is extremely limited, consisting only of isolated case reports. These include a rib chondrosarcoma with intraperitoneal implants treated with peritonectomy and multivisceral resection [6], as well as a case of rib chondrosarcoma with intra-abdominal metastases associated with cytologically confirmed metastatic ascites [7]. The rarity of this presentation prevents the establishment of specific therapeutic recommendations and underscores the importance of documenting new cases.

The objective is to report an uncommon case of peritoneal sarcomatosis secondary to conventional chondrosarcoma and to conduct a critical review of the available literature, emphasizing possible mechanisms of dissemination, diagnostic and therapeutic implications, and the importance of management in specialized sarcoma centers.

## Case presentation

We present the case of a 27-year-old male patient referred to our unit in November 2024 with progressive abdominal enlargement and severe, disabling edema of the left lower extremity. On physical examination, a fixed, indurated, and tender abdominal mass was palpated, measuring 40 x 15 x 15 cm. The lesion extended to both flanks and the left hypochondrium. Massive edema of the ipsilateral lower extremity with a visible superficial collateral venous network was also documented.

**Figure 1:**
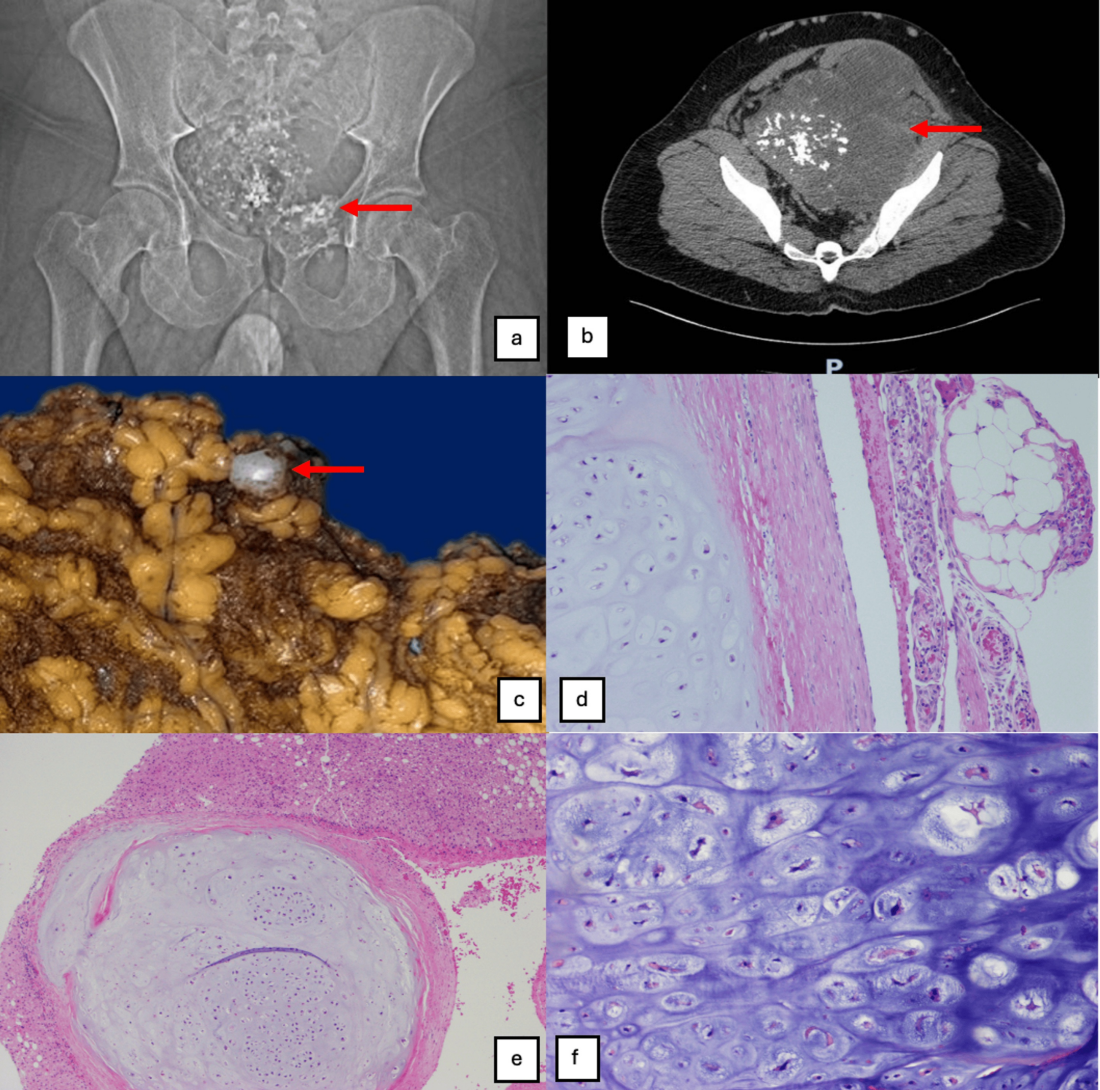
(a) Anteroposterior plain radiograph of the pelvis showing a large centrally located pelvic mass with multiple punctate and amorphous intralesional calcifications, consistent with a chondroid matrix (arrow), occupying a substantial portion of the pelvic cavity. (b) Contrast-enhanced abdominopelvic computed tomography scan (axial view) demonstrating a large, heterogeneous pelvic tumor with extensive calcified chondroid (“popcorn”) matrix and areas of low attenuation suggestive of necrosis (arrow). The mass occupies the pelvis, extends into the abdominal cavity, and displaces adjacent pelvic and abdominal structures. (c) Gross photograph of the omentum showing a grayish, pearly peritoneal tumor implant (arrow). (d) Histological section of the omental implant (hematoxylin and eosin stain, ×20) demonstrating chondroid tumor tissue. (e) Histological section showing a chondrosarcoma nodule in direct contact with the liver parenchyma (hematoxylin and eosin stain, ×10). (f) High-power histological view (hematoxylin and eosin stain, ×40) demonstrating chondroid matrix with moderate cellularity, variably sized lacunae containing more than two chondrocytes, nuclear hyperchromasia, irregular nuclear contours, and focal cellular pleomorphism.

As part of the staging protocol, an anteroposterior plain radiograph of the pelvis was obtained (Figure 1a), revealing a large pelvic mass projecting into the abdominal cavity. The mass was characterized by abundant intralesional punctate and cotton-wool calcifications ("popcorn sign"), with no frank lytic destruction of the iliac bones or ischiopubic rami in this projection, but with a clear mass effect.

A contrast-enhanced abdominal and pelvic computed tomography (CT) scan in axial slices (Figure 1b) confirmed a large, heterogeneous tumor, predominantly in the central pelvis and extending superiorly into the abdomen, measuring more than 20 cm along its longest axis. The mass showed a prominent calcified chondroid matrix, multiple scattered calcified nodules, and hypodense areas suggestive of necrosis. The lesion caused significant displacement of pelvic and abdominal structures, including the bladder and bowel loops, findings highly suggestive of a low-grade chondroid neoplasm. Magnetic resonance imaging (MRI) complemented the study, defining the local extent and confirming the apparent absence of distant metastatic disease at that time.

An image-guided core needle biopsy (Tru-cut) was performed. The preliminary histopathological report indicated a grade 1 (G1) conventional chondrosarcoma. The immunohistochemical profile supported the diagnosis, with diffuse S-100 protein positivity and negative epithelial marker (cytokeratin) staining, ruling out other sarcomas and carcinomas.

The patient was scheduled for oncological resection with curative intent. Given the lesion's extent and the need for secure proximal vascular control, an intraperitoneal approach was chosen. During the exploratory laparotomy, intraoperative findings not evident on initial imaging were identified: spontaneous tumor rupture at the pelvic level with hemorrhagic ascites; peritoneal carcinomatosis: multiple tumor implants were documented in the greater omentum and hepatic visceral peritoneum, resulting in a peritoneal carcinomatosis index of 6; vascular involvement: 180-degree entrapment of the left iliac vessels (artery and vein) by the tumor component, as identified on imaging, and involvement of the left spermatic cord.

Given these findings, primary cytoreductive surgery was performed, including inframesocolic omentectomy, resection of hepatic and peritoneal implants, left orchiectomy, and a type III internal hemipelvectomy (resection of the ischium and pubis while preserving the hip joint and limb), achieving complete macroscopic cytoreduction (CC-0). The patient had a satisfactory postoperative course, maintaining hemodynamic and metabolic stability, and therefore did not require Intensive Care Unit (ICU) management. The patient was transferred to a regular ward and subsequently discharged home after adequate functional recovery.

Histopathological analysis of the complete surgical specimen and peritoneal implants revealed a moderately differentiated (grade 2) conventional chondrosarcoma (Figures 1c-1f), reclassifying the histological grade from the initial biopsy and confirming the malignant nature of the secondary implants. The case was re-evaluated by the multidisciplinary team (MDT). Given the high-risk findings (tumor rupture, histological grade 2, and peritoneal dissemination), adjuvant systemic therapy with immunotherapy and a tyrosine kinase inhibitor was initiated. The patient is currently undergoing the sixth cycle of treatment with good tolerance, remains under close monitoring, and has no clinical or radiological evidence of tumor recurrence to date.

## Discussion

Peritoneal sarcomatosis secondary to chondrosarcoma is an extremely rare condition. Unlike other abdominopelvic sarcomas, chondrosarcoma has a marked predilection for hematogenous dissemination, and peritoneal involvement is an atypical and exceptional presentation [2,5]. Previously reported cases of peritoneal sarcomatosis secondary to chondrosarcoma are extremely limited and are summarized in Table 1*,* including differences in primary tumor location, mechanism of dissemination, treatment strategies, and outcomes. The case reported by González-González et al. describes a rib chondrosarcoma with extensive intraperitoneal dissemination, treated with multivisceral resection and peritonectomy, with subsequent recurrences despite initial complete cytoreduction [6]. The authors suggest that tumor seeding secondary to local recurrences and repeated surgical manipulation may have favored peritoneal dissemination.

**Table 1:** Clinical characteristics and outcomes of reported cases of chondrosarcoma with peritoneal dissemination.

Author (Year)	Age / Sex	Primary Tumor Location	Histological Type	Peritoneal Manifestation	Proposed Mechanism	Treatment	Outcome
González-González et al. (2019) [6]	46/F	6th–8th ribs	Well-differentiated chondrosarcoma	Multiple peritoneal implants (omentum, hepatic capsule, pelvis)	Multiple local recurrences and probable surgical seeding	Multivisceral resection + peritonectomy	Abdominal and thoracic recurrence; death due to disease progression
Smetanina et al. (2022) [7]	38/M	Costal	Chondrosarcoma	Intra-abdominal metastases with malignant ascites (cytological confirmation)	Hematogenous dissemination with secondary peritoneal involvement	Palliative management	Disease progression
Present case	27/M	Pelvis (pubis/ilium)	Conventional chondrosarcoma, G2	Extensive peritoneal sarcomatosis (omentum, hepatic capsule, hepatorenal recess)	Spontaneous tumor rupture	Complete cytoreduction (hemipelvectomy + omentectomy + resection of implants)	Early follow-up (no evidence of recurrence at the time of reporting)

Additionally, Smetanina et al. reported a patient with rib chondrosarcoma who developed intra-abdominal metastases with cytological confirmation of tumor cells in the peritoneal fluid, accompanied by metastatic ascites [7]. This finding broadens the clinical spectrum of peritoneal dissemination in chondrosarcoma and demonstrates that, in addition to macroscopic implants, the disease can manifest as malignant ascites, with important diagnostic implications. In this context, ascitic fluid cytology can be a useful diagnostic tool in patients with a history of chondrosarcoma and atypical abdominal presentation.

In the present case, intraoperative identification of multiple peritoneal implants and documentation of spontaneous tumor rupture support the hypothesis of dissemination via capsular violation, a well-recognized mechanism in peritoneal sarcomatosis associated with other sarcomas [4]. This pattern suggests that, although infrequent, peritoneal dissemination in chondrosarcoma can occur in locally advanced disease or after loss of tumor integrity.

From a therapeutic standpoint, there are no specific guidelines for managing peritoneal sarcomatosis secondary to chondrosarcoma. Current recommendations are derived from a heterogeneous series of peritoneal sarcomatosis due to soft tissue sarcomas and emphasize that complete cytoreductive surgery, performed in high-volume centers with appropriate patient selection, is the mainstay of therapy for locoregional control [4,8]. However, the benefit of Hyperthermic IntraPEritoneal Chemotherapy (HIPEC) and systemic chemotherapy in chondrosarcoma remains uncertain, largely due to the well-known intrinsic chemoresistance of this tumor [1,2].

Recent advances in the molecular characterization of chondrosarcoma have improved understanding of the mechanisms of tumor progression, although their therapeutic impact in advanced metastatic disease remains limited [1,9].

## Conclusions

Peritoneal sarcomatosis associated with chondrosarcoma is a rare and likely underdiagnosed entity. Available evidence consists of isolated case reports, including presentations with macroscopic peritoneal implants and metastatic ascites. This case reinforces the need to consider atypical patterns of dissemination in patients with advanced or recurrent chondrosarcoma and underscores the importance of management at specialized sarcoma centers with experience in complex surgery and close follow-up. Systematic documentation of new cases is essential to advance the biological and therapeutic understanding of this unusual clinical presentation.
